# Preexisting vaccine-primed heterosubtypic T cell immunity protects the maternal-fetal unit from adverse influenza outcomes in mice

**DOI:** 10.1172/JCI179230

**Published:** 2025-01-02

**Authors:** Valeria Flores Malavet, Kunal Dhume, Ali Satchmei, Andrea C. Arvelo, Aaron J. Beaird, Siva N. Annamalai, Lauren A. Kimball, K. Kai McKinstry, Tara M. Strutt

**Affiliations:** Immunity and Pathogenesis Division, Burnett School of Biomedical Sciences, College of Medicine, University of Central Florida, Orlando, Florida, USA.

**Keywords:** Immunology, Infectious disease, Cellular immune response, Influenza, Th1 response

## Abstract

The risk of severe outcomes of influenza increases during pregnancy. Whether vaccine-induced T cell memory–primed prepregnancy retains the ability to mediate protection during pregnancy, when systemic levels of several hormones with putative immunomodulatory functions are increased, is unknown. Here, using murine adoptive transfer systems and a translationally relevant model of cold-adapted live-attenuated influenza A virus vaccination, we show that preexisting virus-specific memory T cell responses are largely unaltered and highly protective against heterotypic viral challenges during pregnancy. Expression of the transcription factor T-bet, which is upregulated in antiviral T cells responding in pregnant mice, is critical in preventing hormone-associated gain of detrimental T helper type 2 (T_H_2) attributes reported in other settings. Beyond antiviral effects, preexisting vaccine-primed T cell immunity prevents metabolic dysfunction in gravid dams and adverse neonatal outcomes often associated with maternal influenza infection. These results demonstrate robust protection of the maternal-fetal unit from severe consequences of respiratory virus infection by preexisting T cell immunity.

## Abstract 2

Findings in the study show that pre-exisiting memory T cell immunity against influenza induced by vaccination with live-attenuated vaccines prior to pregnacy safely protects the maternal-fetal unit from the adverse outcomes of maternal influenza A virus infection.

## Introduction

Pregnant women and their developing infants suffer disproportionally high morbidity and mortality from influenza-related complications (1). Changes in cardiovascular physiology (2), the immune system (3), low vaccine efficacy (4), and a reluctance to be vaccinated (5, 6) all increase the likelihood that exposure to respiratory viruses during pregnancy will result in serious infection. In recent years, increasing proportions of expectant mothers suffering from influenza have required hospitalization (7). Likewise, the odds of requiring critical care, adverse fetal outcomes, and death following development of COVID-19 increase during pregnancy (8).

While vaccination is the best means to induce protective immunity against influenza A virus (IAV), vaccines must be administered with caution during pregnancy (9). Only inactivated IAV vaccines that target the generation of hemagglutinin and neuraminidase-specific antibodies (Abs) are approved for use (10, 11). Inactivated vaccine administration at any trimester of pregnancy is considered safe and effective at preventing seasonal IAV infection (12–15). The neutralizing Abs induced by such vaccines have minimal efficacy, however, in preventing infection with drifted or emergent pandemic strains of IAV (9). Abs that target conserved stem regions of hemagglutinin (16) and T cell responses specific for conserved internal viral proteins, such as those induced by live attenuated cold-adapted IAV vaccines (LAIV), can protect against drifted and/or shifted strains of the virus, respectively (17–21). Means to induce universal protection against IAV in pregnant women through vaccination are lacking as LAIV use is contraindicated during pregnancy (11). T cell responses against the conserved internal IAV proteins, commonly referred to as heterosubtypic immunity, can persist and protect against IAV for 9 to 12 months (22–24). Therefore, if induced by vaccination prior to conception, heterosubtypic T cell responses against IAV could potentially protect expectant mothers against pandemic threats throughout gestation. However, whether and how pregnancy impacts the persistence and protective potential of preexisting IAV-specific immunity is not well established.

Beyond tolerance mechanisms at the maternal-fetal interface (25, 26), alterations in the frequency and functional capacity of immune cells essential for controlling respiratory infections occur during pregnancy (27–29). Pregnancy hormone–associated suppression of immunity (30, 31) and compensatory increases in innate immunity result in heightened immunopathology during infection (3, 31–34). CD4^+^ T cell polarization biases driven by increased levels of pregnancy-associated hormones also interfere with effective antiviral immunity (35–38). The consensus is that pregnancy biases CD4^+^ T cell responses toward T helper type 2 (T_H_2) humoral immune responses, thus weakening antiviral T_H_1 cellular responses (39, 40). While debated (41, 42), this view fits the broader paradigm that cellular responses that could harm the developing fetus are restricted during pregnancy (43–45).

Here, we use well-characterized murine adoptive transfer and LAIV prime and challenge models of IAV infection to address whether pregnancy hinders the ability of memory CD4^+^ T cells to mediate protection against IAV. We show that,when recalled by infection during pregnancy, IAV-specific memory CD4^+^ T cells retain protective antiviral T_H_1 characteristics. Unexpectedly, primary antiviral T_H_1 CD4^+^ T cell responses are also, for the most part, unaltered. Mechanistically, rather than being decreased, primary IAV-specific effector CD4^+^ T cells express enhanced levels of the master T_H_1-associated transcription factor T-bet during pregnancy. Only when CD4^+^ T cells responding against IAV are deficient in T-bet and thus poised to produce T_H_2 cytokines (46, 47), are enhanced T_H_2 functional response profiles generated during pregnancy. Finally, in agreement with the latter findings, LAIV vaccination prior to pregnancy, which mirrors the scenario of vaccination of females attempting to conceive, induces a memory T cell population that persists and protects the maternal-fetal unit from the severe consequences of IAV infection.

## Results

### Antiviral CD4^+^ T cell functions are retained during pregnancy.

**Figure 1:**
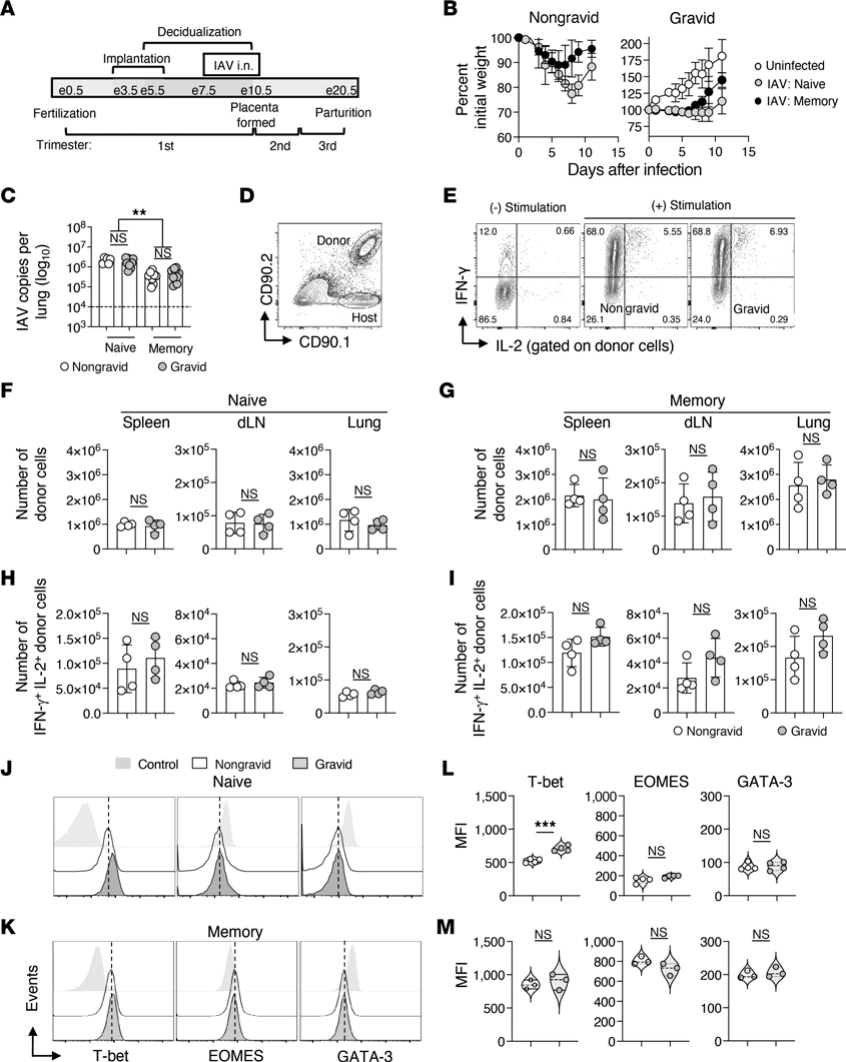
Naive and memory CD4^+^ T cell responses against IAV are unaltered during pregnancy. Congenic HNT CD4^+^ TcR Tg naive or memory CD4^+^ T cells, 3 × 10^6^, were adoptively transferred to unprimed nongravid or timed-pregnant gravid BALB/c female recipients subsequently infected with 0.5 LD_50_ PR8. (**A**) Timeline showing the trimester and embryonic day (e) gravid mice were infected and (**B**) morbidity following infection. (**C**) Lung viral titers evaluated on 4 days post infection (dpi) (summation of 2 separate experiments with *n* = 4 per group). On 7 dpi, donor responses in spleen, draining lymph nodes (dLN), and lung were characterized by flow cytometry and ICCS. Representative staining is shown in **D** and **E**. Enumeration of donor cells and dual IFN-γ and IL-2 cytokine production for naive and memory cells (**F**–**I**). Representative T-bet, EOMES, and GATA-3 expression and corresponding MFI for naive (**J** and **L**) and memory (**K** and **M**) derived donor cells in the lung 7 dpi (*n* = 3–4 per group, representative of 2 separate experiments). Staining controls in (**J** and **K**) are naive CD4^+^ T cells. Ordinary 1-way ANOVA with Tukey’s multiple comparison post test was used in **A** and Students *t* test in **F**–**M**.

IAV-specific memory CD4^+^ T cells can mediate protection against IAV (48). To interrogate whether the functions of IAV-specific memory T cell are altered during pregnancy, we first employed an adoptive transfer model in which naive and resting memory IAV-specific CD4^+^ T cells were transferred separately to nonpregnant female (nongravid) and timed-pregnant (gravid) recipient hosts. Donor cells were transferred 1 week after the disbanding of mating cages. As shown in Figure 1A, for gravid mice, this timepoint coincided with the transition between the first and second trimester of murine pregnancy (49). Memory cells, 3 × 10^6^, a number previously determined to be capable of mediating protection against lethal infection (50, 51), or a comparable number of naive cells, were transferred. Recipient hosts were infected with a pathogenic 0.5 LD_50_ dose of PR8 (H1N1) virus on the same day.

Nongravid and gravid IAV-infected recipients were monitored for morbidity evaluated by weight loss. IAV-specific memory cells protected nongravid females from the substantial weight loss associated with this dose of PR8 (Figure 1B). Gravid female weight loss after infection was masked by pregnancy-associated weight gain; however, recipients of IAV-specific memory cells gained more weight than naive-cell recipients, but less than uninfected, gestational, and age-matched gravid dams (Figure 1B). In separate experiments, peak lung viral titers were evaluated. Nongravid recipients of IAV-specific memory CD4^+^ T cells had reduced lung viral titers versus naive recipients 4 days after infection (Figure 1C). Comparable memory CD4^+^ T cell–mediated control of virus was evident in gravid recipients. These findings support that protective antiviral effector functions of memory CD4^+^ T cells are not suppressed by the pregnancy environment.

We next characterized the magnitude and functional responses of the transferred cells in the spleen, draining lymph nodes (dLN), and lung 7 days after infection. Representative gating and staining for intracellular cytokines are shown in Figure 1, D and E. Neither the magnitude of the response (Figure 1, F and G) nor the T_H_1-associated (IFN-γ, IL-2, and TNF) multicytokine producing capacity of naive or memory IAV-specific CD4^+^ T cells derived effectors in the spleen, dLN, and lung differed between nongravid and gravid hosts (Figure 1, H and I, and Supplemental Figure 1; supplemental material available online with this article; https://doi.org/10.1172/JCI179230DS1).

T_H_2-associated cytokine production was also evaluated. As anticipated for primary antiviral responses, the frequency of IL-4–, IL-5–, and IL-13–producing cells derived from naive donors was low to undetectable in the spleen, dLNs, and lungs in nongravid hosts (Supplemental Figure 2, A and B, left panel). Similar results were seen in gravid hosts, and when memory CD4^+^ T cell responses were compared between nongravid and gravid females (Supplemental Figure 2B, right panel). Substantial IL-4, IL-5, and IL-13 production (Supplemental Figure 2, C and D), but not IFN-γ or IL-17 (Supplemental Figure 2, E and F), was readily detectable, however, when a control population of T_H_2 CD4^+^ T effector cells responding to PR8 in separate nongravid and gravid hosts was evaluated.

We next characterized expression of the transcription factors T-bet, EOMES, and GATA-3. The mean fluorescent intensity (MFI) of T-bet expressed by lung effectors derived from naive donor cells was higher in gravid than nongravid hosts (Figure 1, J and L). A similar increase in T-bet MFI was seen in endogenous lung CD44^hi^ CD4^+^ and CD8^+^ T cells 7 days after IAV infection (Supplemental Figure 3A). Endogenous CD8^+^, but not CD4^+^, T cells responding against IAV in gravid mice also expressed increased EOMES (Supplemental Figure 3A). No other differences in transcription factor MFI were evident (Figure 1, J–M, and Supplemental Figure 3A). Collectively, the absence of detectable shifts away from T_H_1-associated cytokine production by donor, as well as endogenous T cells (Supplemental Figure 3B), and retention of T_H_1 transcription factor expression support that the recall of preexisting IAV-specific memory CD4^+^ T cells is not altered by the pregnancy environment.

### T_H_1-polarized CD4^+^ T cell effector IFN-γ production is unaltered by pregnancy levels of estradiol.

Unexpectedly, our in vivo observations supported that both primary and secondary antiviral CD4^+^ T cell responses were not altered during pregnancy. Suppressed responses and/or shifts toward T_H_2 characteristics were not evident in at least 6 experiments. A reproducible and marked 10%–25% increase in IL-4–, IL-5–, and IL-13–producing cells was, nonetheless, observed when positive controls for intracellular staining, T_H_2 effector CD4^+^ T cells, were transferred to gravid recipients infected with IAV (Supplemental Figure 2, C and D). The latter finding favors the hypothesis that the pregnancy environment enhances IL-4–, IL-5–, and IL-13–production from cells poised to produce these cytokines rather than causing shifts in T_H_1 polarization toward the T_H_2 pole.

**Figure 2:**
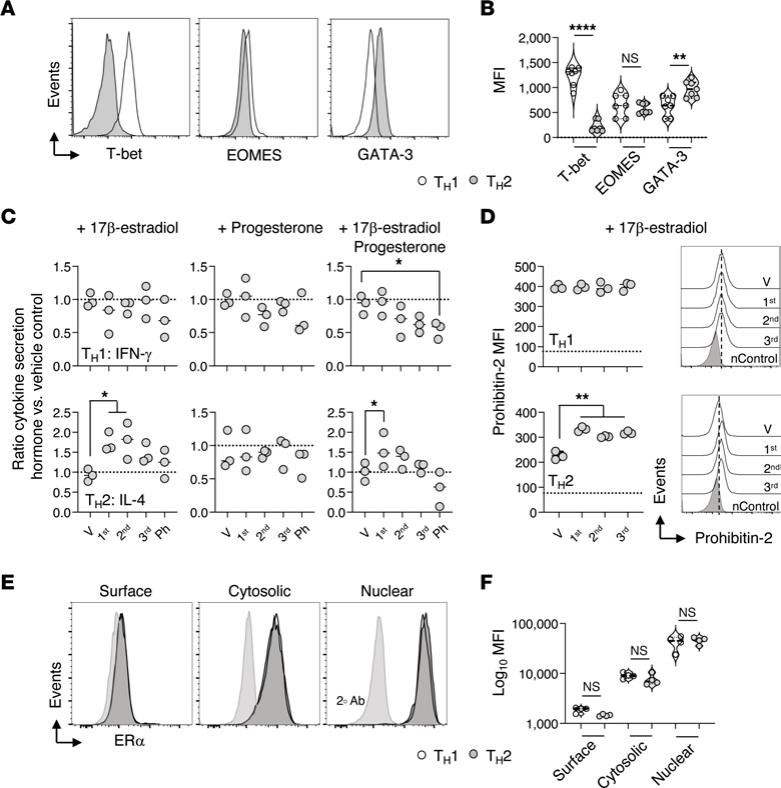
T_H_1 effector IFN-γ production is unaltered by pregnancy levels of estradiol. Naive HNT CD4^+^ T cells were cultured in triplicate for 4 days with APC and peptide under T_H_1- or T_H_2-polarizing conditions. Media was supplemented with 17-β estradiol (E2), progesterone (P4), 17β-estradiol plus progesterone, or vehicle control (V) at concentrations comparable to those found systemically during the 1st (0.0176 μM E2 and 0.096 μM P4), 2nd (0.0353 μM E2 and 0.297 μM P4), and 3rd (0.0053 μM E2 and 0.382 μM P4) trimesters of pregnancy or administered pharmaceutically (Ph) (1 μM of E2 and P4). T-bet, EOMES, and GATA-3 transcription factor expression (**A**–**C**) ratio of T_H_1 IFN-γ (top row) and T_H_2 IL-4 (bottom row) cytokine secretion following culture with pregnancy hormones versus vehicle supplemented media. (**D**) MFI and representative histograms of Prohibitin-2 expression by T_H_1 and T_H_2 effectors cultured with and without supplemental 17-β estradiol. Naive (n) cell expression of Prohibitin-2 is shown as dashed line in graphs and nControl histograms. (**E** and **F**). Expression of estrogen receptor α (ERα) visualized with indirect surface, intracellular, and nuclear staining of polarized cells. Fluorescent-conjugated secondary Ab only control (2°Ab) histograms are indicated in **E**. Data representative of 3 replicate experiments. Students *t*- tests were used for pairwise comparisons in **A** and **F**, and ordinary 1-way ANOVA with Dunnett’s multiple comparison post test used in **C** and **D**.

To test this, purified naive CD4^+^ T cells were cultured under T_H_1 or T_H_2-polarizing conditions in vehicle or estrogen and/or progesterone-supplemented media for 4 days. Effector cell cytokine secretion into supernatants following anti-CD3 Ab-mediated TcR restimulation was evaluated by ELISA. T_H_1 and T_H_2 polarization was also confirmed by characteristic transcription factor expression (Figure 2, A and B). In agreement with our in vivo observations, IFN-γ production by T_H_1 effectors was unaltered by the presence of pregnancy hormones at concentrations comparable to those found systemically during pregnancy (52) (Figure 2C, top row). When both hormones were present, higher concentrations suppressed IFN-γ production by T_H_1 cells. IL-4 secretion by T_H_1 effectors was below the limit of detection regardless of pregnancy hormone supplementation (data not shown). In contrast to the unaltered T_H_1 effector responses, T_H_2 effector cells cultured in the presence of supplemental estradiol produced more IL-4 than cells cultured with vehicle alone (Figure 2C, bottom row).

Expression of Prohibitin-2, an estrogen response gene that participates in the regulation of estrogen signaling pathways in CD4^+^ T cells (53, 54), was also evaluated. Expression of Prohibitin-2 was increased in T_H_2, but not T_H_1, effector cells cultured with supplemental estradiol (Figure 2D). While indirect effects of pregnancy hormones on antigen-presenting cells (APCs) within the polarizing cultures may also contribute, these findings support that T_H_1 and T_H_2 effector cells possess the potential to differentially respond to estradiol.

As CD4^+^ T cells preferentially express estrogen receptor α over β (55), estrogen receptor α expression by T_H_1 versus T_H_2 cells was assessed next. Comparable levels of expression by both CD4^+^ T cell subsets (Figure 2, E and F) further supports that alternative differences in the effectors, driven either by direct or indirect estrogen-mediated signals, contribute to the ability of estrogen to increase IL-4–, IL-5–, and IL-13–production from T_H_2 but not T_H_1 cells.

### T-bet insulates antiviral CD4^+^ T cells from the influences of the pregnancy environment.

To test whether CD4^+^ T cells need to be poised for T_H_2 cytokine production to be altered by the pregnancy environment during IAV infection, T-bet–deficient mice were employed. T-bet–deficient T cells responding against IAV retain the ability to produce IFN-γ but show increased T_H_2 characteristics because negative regulatory mechanisms controlling GATA-3 are lacking (56, 57). Nevertheless, T-bet–deficient CD4^+^ T cells protect against IAV (47, 58), in part because of the actions of the paralog transcription factor EOMES (47, 59, 60). We therefore asked whether antiviral T-bet–deficient immune responses, which are protective — though prone to produce T_H_2 cytokines in nongravid mice — are altered by the pregnancy environment.

**Figure 3:**
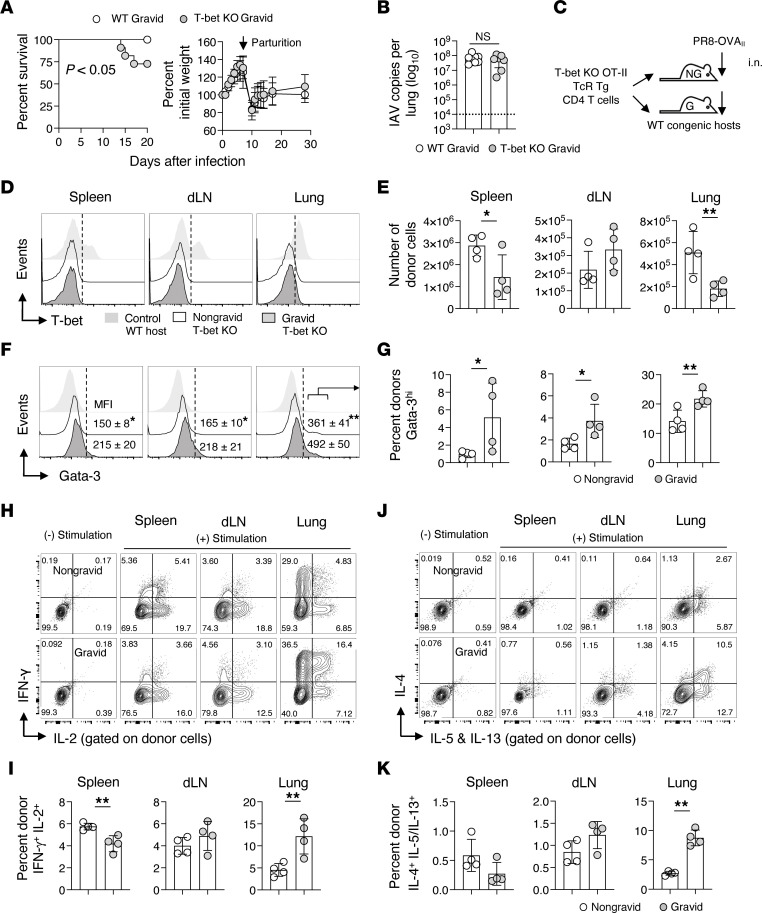
T-bet–deficient immunity is altered during pregnancy and fails to protect the maternal-fetal unit from the adverse outcomes of IAV. C57BL/6J WT and T-bet–deficient (KO) timed-pregnant gravid mice were infected with 0.5 LD_50_ PR8 at the transition between the first and second trimester of pregnancy and (**A**) survival and morbidity, and (**B**) lung viral titers on 7 dpi were monitored (representative of 2 independent experiments with *n* = 5–6 per group per experiment). Parturition is marked with an arrow. In separate experiments, 2 × 10^6^ naive congenic T-bet–deficient OT-II CD4^+^ TcR Tg cells were transferred to nongravid (NG) or timed-pregnant gravid (**G**) WT C57BL/6J recipients that were subsequently infected as described above with 0.2 LD_50_ PR8-OVA_II_ (**C**). On 7 dpi, donor responses in the spleen, dLN, and lung were characterized by flow cytometry and ICCS (*n* = 4 per group, representative of 2 separate experiments). Expression of (**D**) T-bet, and (**E**) enumeration of donor cells. Control histograms in (**D**) are WT host T cells. (**F**) GATA-3^hi^ expression by donor cells and (**G**) frequency of GATA-3^hi^ expressing donor cells in all organs. Representative donor cell ICCS staining and enumeration of dual IFN-γ and IL-2 and IL-4– and IL-5–and IL-13–producing cells in all organs (**H**–**K**). The Log Rank test was used in **A**, and Students *t* test for pairwise comparisons in **B**, **E**, **G**, **I**, and **K**.

Timed-pregnant WT and T-bet–deficient mice were infected with 0.5 LD_50_ PR8 at the transition between the first and second trimesters of pregnancy, and morbidity, mortality, lung viral burdens, and litter outcomes following parturition were monitored. While gravid T-bet deficient dams were indistinguishable from WT dams in terms of weight gain following infection, their survival was reduced (Figure 3A). Control of lung viral titers was, however, comparable between the 2 genotypes (Figure 3B). Though litter survival was similar following parturition, pups born to surviving T-bet–deficient versus WT dams were impaired in postnatal weight gain (Supplemental Figure 4, A and B). These findings suggest that T-bet expression is critical for the generation of antiviral immune responses capable of protecting the maternal-fetal unit from the adverse outcomes of IAV infection.

Given that numerous cells that participate in immune responses against IAV express T-bet (61), we next restricted T-bet deficiency to CD4^+^ T cells. Naive OT-II T-bet–deficient CD4^+^ T cells were transferred to nongravid and gravid T-bet–sufficient mice at the transition between the first and second trimester of pregnancy (Figure 3C). Mice were then infected with 0.2 LD_50_ PR8-OVA_II_. Lack of T-bet expression by donor cells and expression by T-bet–sufficient host cells was verified by flow cytometry 7 days after infection (Figure 3D). The effect of pregnancy on donor antiviral responses against IAV was then characterized.

The recovery of T-bet–deficient donor cells was reduced in the spleens and lungs, but not dLNs, of gravid recipients 7 days after infection (Figure 3E). When T-bet–deficient donor GATA-3 expression was analyzed, the MFI was higher in gravid versus nongravid hosts (Figure 3F). Since increased GATA-3 expression is associated with T_H_2 responses (62, 63) and T_H_1 and T_H_2 cells differ in their expression of chemokine receptors (64, 65), it is likely that differential migration contributes to the reduced numbers of donors observed in the lung and spleen of gravid hosts. Further comparisons are therefore presented as frequencies within the donor CD4^+^ T cell population present. Increased frequencies of GATA-3^hi^ T-bet deficient donors cells were found in the spleen, dLN, and lung of gravid versus nongravid recipients (Figure 3G).

Donor multicytokine production day 7 after infection was evaluated next. T-bet–deficient effector CD4^+^ T cells in nongravid recipients retained substantial IFN-γ production (Figure 3H). T-bet–deficient effector CD4^+^ T cells responding in gravid recipients also produced IFN-γ and displayed increased IFN-γ, IL-2, and TNF multicytokine-producing capacity, especially in the lung (Figure 3, H and I, and Supplemental Figure 4C). When T_H_2 cytokine production was analyzed, IL-4–, IL-5–, and IL-13–producing capacity was evident in all organs and was increased in the lungs of gravid versus nongravid hosts (Figure 3, J and K, and Supplemental Figure 4D). Thus, the Th2 cytokine–producing potential of antiviral CD4^+^ T cells responding against IAV is enhanced during pregnancy, but only when T-bet is absent.

### Persistence and recall of heterosubtypic T cell immunity induced by LAIV is unhindered during pregnancy.

**Figure 4:**
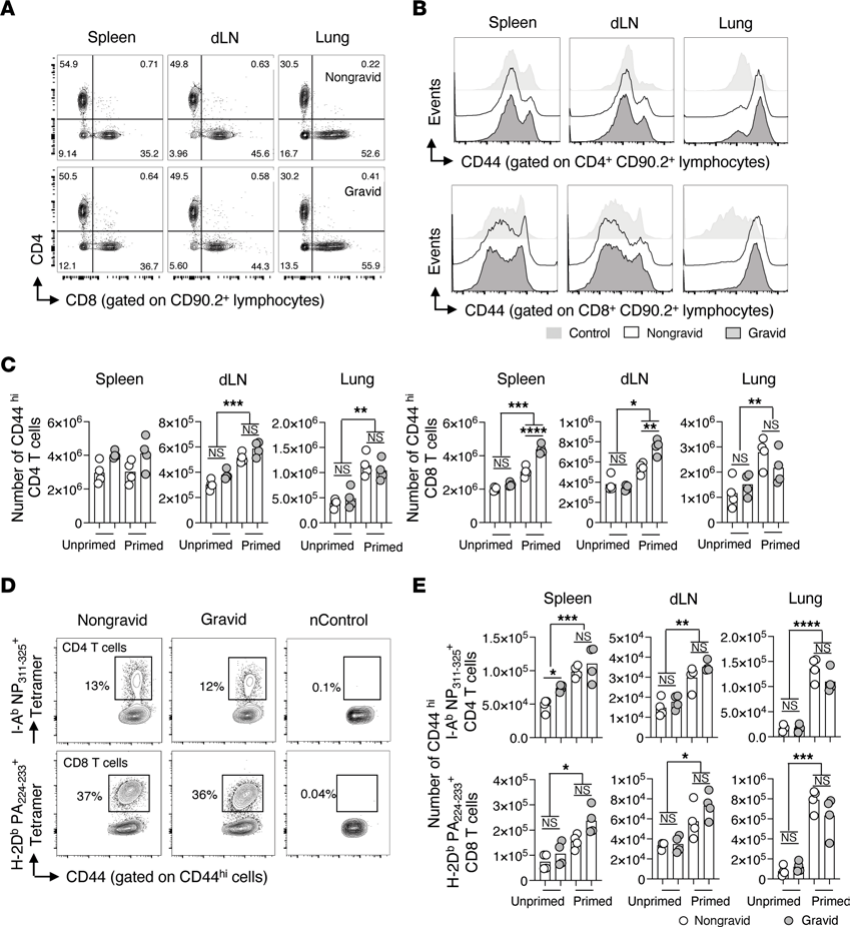
LAIV-primed heterosubtypic T cell recall responses against IAV are retained in pregnant mice. Groups of C57BL/6J female mice were unprimed or primed with 2,500 TCID_50_ of LAIV. Timed pregnancies were established within each group. At day 30 after priming, mice were challenged with 0.5 LD_50_ PR8. At this timepoint, gravid dams were at the transition between the first and second trimester of pregnancy. On day 7 after challenge, CD4^+^ and CD8^+^ T cell responses were characterized by flow cytometry and IAV-specific cells in spleens, dLNs, and lungs visualized with tetramer staining. Representative (**A**) frequencies of CD4^+^ and CD8^+^ T cells in nongravid and gravid mice, and (**B**) CD44 expression on CD4^+^ and CD8^+^ T cells. Enumeration (**C**) of CD44^hi^ CD4^+^ and CD8^+^ T cells. Representative (**D**) tetramer staining and (**E**) enumeration of IAV-specific T cells in unprimed and primed mice (*n* = 4 mice per group, representative of 3 separate experiments). Uninfected animals were used as naive (n) controls. Ordinary 1-way ANOVA with Šidák’s multiple comparison post test was used in **C** and **E**.

We next asked whether endogenous T cell memory induced by vaccination before pregnancy persists and retains protective antiviral functions during pregnancy. To test this, a translationally relevant model of LAIV priming followed by heterotypic IAV challenge was employed. Female mice were primed with LAIV or treated with PBS intranasally (i.n.) at least 21 days prior to the initiation of timed pregnancies. At time points 30 days or greater after LAIV (H3N2) priming, and when gravid dams were at the transition between the first and second trimester of pregnancy, unprimed and primed nongravid and gravid mice were challenged with a pathogenic dose of PR8 (H1N1) virus and CD4^+^ and CD8^+^ T cell responses in the spleen, dLN, and lung were evaluated. On day 6 after challenge, when all gravid dams were still pregnant, differences in total CD4^+^ or CD8^+^ T cell frequency and number were not evident (Figure 4A and data not shown). Numbers of activated CD44^hi^ CD4^+^ and CD8^+^ T cells were increased in LAIV-primed mice regardless of pregnancy status (Figure 4, B and C). When T cell responses in nongravid and gravid mice were compared, only total numbers of CD44^hi^ CD8^+^ T cells were higher in the secondary lymphoid organs of LAIV-primed gravid females (Figure 4C). Interestingly, this subset of immune cells is also found at higher frequency in human studies of pregnancy (66, 67).

We next enumerated IAV-specific CD4^+^ and CD8^+^ T cell responses by tetramer staining and flow cytometry analysis (Figure 4, D and E). Increased numbers of IAV-specific CD4^+^ and CD8^+^ T cells were present in all organs of all LAIV-primed versus unprimed animals (Figure 4E). Importantly, total numbers of IAV-specific T cells in the spleens, dLNs, and lungs of LAIV-primed nongravid and gravid mice were comparable, although a modest but significant increase in the number of IAV-specific CD4^+^ T cells in the spleens of gravid versus nongravid unprimed mice was noted.

### Heterosubtypic T cell immunity induced by LAIV is protective during pregnancy.

**Figure 5:**
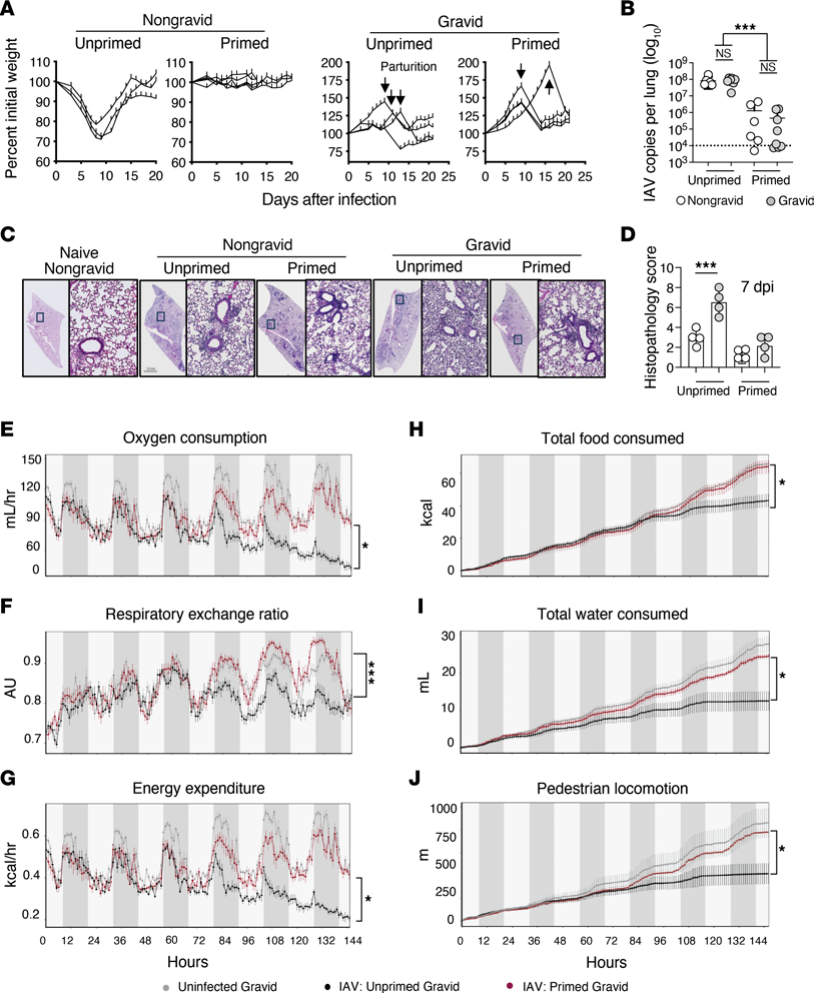
LAIV priming protects pregnant mice against pathogenic IAV infection and preserves maternal metabolic wellness. Groups of C57BL/6J female mice were unprimed or primed with 2,500 TCID_50_ LAIV and timed-pregnant and nongravid controls established within each group. At day 30 after priming, mice were challenged with 0.5 LD_50_ PR8 as described in the legend of Figure 4 and (**A**) morbidity was monitored (*n* = 3–4 mice per group, representative of 2 separate experiments). Parturition is marked with arrows. On day 7 after challenge, (**B**) viral titers and (**C** and **D**) immunopathology in H & E-stained lung sections was determined (*n* = 4–8 mice per group, representative of 2 separate experiments). Representative images in (**C**) are stitched 20 × magnification Z-stacks of whole lung sections and the corresponding 7 × zoom of indicated regions. Following infection, indirect calorimetry parameters were monitored with the Promethion Cage Monitoring system and hourly (**E**–**G**) and cumulative data (**H**–**J**) are shown (*n* = 4 mice per group, representative of 2 separate experiments). In **H**, uninfected and LAIV-primed datasets are overlapping. Ordinary 1-way ANOVA with Šidák’s multiple comparison post test was used in **B** and **D**. ANCOVA tests on raw data in **E**–**J** were performed by CALR.

Given the similarity in the T cell responses above, we next assessed the capacity of LAIV priming prior to pregnancy to prevent IAV infection–associated morbidity. In agreement with the detection of increased numbers of activated IAV-specific T cells, LAIV priming of mice prevented the weight loss associated with IAV challenge of nongravid females (Figure 5A). The weight gain of pregnancy masks IAV-associated weight loss in gravid females until parturition, limiting the ability of this approach to evaluate the protective nature of maternal heterosubtypic immunity. However, when weight gain following IAV challenge was compared, LAIV-primed gravid dams gained more weight than unprimed gravid dams with and without normalization to the number of viable pups born (*P* < 0.001 and *P* *<* 0.01, 2-tailed Students *t* test, respectively); LAIV-primed gravid dam weight gain matched uninfected, age and gestational age-matched controls (data not shown).

Control of virus and lung histopathology following heterotypic IAV challenge were evaluated next. On day 7 after infection, control of the virus in the lung was comparable between nongravid and gravid LAIV-primed mice (Figure 5B). In agreement with previous studies (33, 34, 68) and the increased severity of IAV infection in pregnant females, lung histopathology scores were markedly higher for unprimed gravid females than for nongravid females 7 days after challenge (Figure 5, C and D). Importantly, LAIV priming prior to pregnancy prevented the pronounced lung histopathology associated with maternal IAV infection, and histopathology scores between primed nongravid and gravid mice were similar (Figure 5, C and D).

A focus only on maternal control of virus and lung histopathology may not reveal all outcomes of the recall of LAIV-induced heterosubtypic immunity on the maternal-fetal unit. As nutritional wellness is essential during pregnancy (69), we next asked whether LAIV-induced heterosubtypic immunity protects maternal metabolic health following IAV infection. Metabolic health was evaluated by indirect calorimetry in which oxygen consumption, respiratory exchange ratio, energy expenditure, food consumption, water intake, and physical activity were continuously monitored for 6 days following challenge infection (70). Beginning 4-days after challenge, oxygen consumption, respiratory exchange ratios, and the energy expenditure readings for unprimed gravid females began to decline compared with LAIV-primed gravid dams and uninfected gravid female controls (Figure 5, E–G). Likewise, the cumulative amount of food and water intake by LAIV-primed gravid females during the first 6 days of the challenge infection was higher than that of unprimed gravid females (Figure 5, H and I) and was almost indistinguishable from uninfected gestation-age–matched gravid dams. Finally, differences in physical activity, measured by total pedestrian locomotion, were evident (Figure 5J) with unprimed gravid females displaying markedly less physical activity than LAIV-primed gravid females. Collectively, these observations support that LAIV priming of heterosubtypic T cell immunity prior to pregnancy can protect metabolic and nutritional wellness following maternal IAV infection.

### Heterosubtypic T cell immunity protects against the adverse fetal outcomes of maternal IAV infection.

We next evaluated whether LAIV priming prior to pregnancy can prevent the adverse fetal outcomes associated with maternal IAV infection (71, 72). Female mice were primed with LAIV (H3N2) or treated with PBS i.n. at least 21 days before the generation of timed pregnancies. At time points of 30 days or greater after priming, unprimed and primed mice were challenged with PR8 (H1N1). Gravid dams were at the transition between the first and second trimester of pregnancy when challenged. Following parturition, litter survival, birth weight, and pup development were monitored. To not compromise litter survival, dams and litters were undisturbed for 72 hours following parturition and 3 postnatal-day pup weights were used in lieu of birth weight.

**Figure 6:**
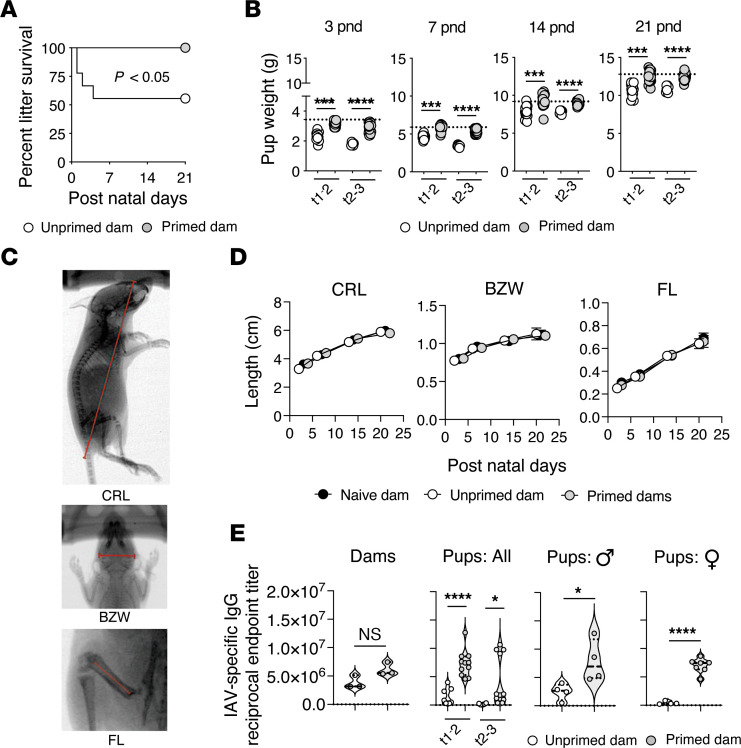
LAIV-primed heterosubtypic immunity protects against the detrimental fetal outcomes of maternal IAV infection. Groups of BALB/c female mice were unprimed or primed with 2,500 TCID_50_ LAIV and timed pregnancies were established. On 30 days after priming, when gravid dams were at the transition between the first and second trimester of pregnancy (t1–2) (**A**–**E**) or, alternatively at the second and third trimester of pregnancy (t2–3) (**B** and **E**), unprimed and LAIV-primed gravid mice were challenged with 0.5 LD_50_ PR8. Following parturition, (**A**) litter survival was monitored. On the indicated postnatal days, (**B**) pup weight, (**C** and **D**) crown-rump length (CRL), bizygomatic width (BW), and femur length (FL) were assessed (*n* = 4 dams per group and *n* = 6–20 pups per group, representative of 2 separate experiments). On postnatal day 21, serum from dams and sexed pups was collected and (**E**) PR8-specific Ab titers were determined (*n* = 3 dams per group and *n* = 4–7 sexed pups per group, representative of 2 separate experiments). Naive, uninfected dam and pups Ab titers are shown as dashed lines. The Log Rank test was used in **A** and Student’s *t* test for pairwise comparisons shown in **B** and **E**.

The survival of litters born to unprimed dams maternally infected with IAV was lower than that of litters born to LAIV-primed dams (Figure 6A). Further evaluation of litters revealed that pups born to unprimed dams infected with IAV at the transition between the first and second trimester of pregnancy had lower 3-day postnatal weights than pups born to uninfected dams (the average is represented as a dashed line, *P* <0.001, 2-tailed Student’s *t*-test) (Figure 6B) recapitulating offspring outcomes observed clinically following maternal IAV infection (71, 72). The low postnatal weights of pups born to unprimed dams remained evident to 21 postnatal days and is consistent with unprimed gravid dams having lower food and water intake following maternal IAV infection. In contrast, the postnatal weights of pups born to LAIV-primed dams challenged with PR8 were not different from pups born to uninfected dams (Figure 6B). Similar outcomes were observed when gravid dams were challenged with heterotypic IAV at the transition between the second and third trimester of pregnancy (Figure 6B).

To ascertain whether the differences in pup weight discussed above were caused by infection associated impacts on morphological development, X-ray images of pups were taken at 3, 7, 14, and 21 postnatal days. Standard measures of morphological size, including crown-rump length, bizygomatic width, and femur lengths were then assessed. Differences in morphological development were not evident at any postnatal timepoint between pups born to unprimed and LAIV-primed females challenged maternally with heterotypic IAV (Figure 6, C and D). These findings suggest that the low post natal weights observed in pups born to unprimed dams after IAV infection was the result of lower body mass.

Finally, the impact of LAIV (H3N2)–primed immunity on the transplacental transfer of maternal virus-specific IgG to offspring following PR8 infection at the transition between the first and second trimester of pregnancy was determined. On day 30 after PR8 challenge, PR8-specific–IgG convalescent serum Ab titers in unprimed and LAIV-primed dams were comparable (Figure 6E, far left panel). In contrast, titers of maternally transferred PR8-specific IgG at 21 postnatal days were higher in the sera of LAIV-primed dam offspring than in the sera of unprimed dam offspring (Figure 6E, mid-left panel). Similar differences in maternally transferred PR8-specific IgG Ab titers at 21 postnatal days were evident when gravid dams were challenged with PR8 at the transition between the second and third trimesters of pregnancy. When male and female pups were analyzed separately, differences in maternally transferred-PR8–specific IgG titers remained evident (Figure 6E, right panels). Thus, in addition to protecting the maternal-fetal unit from the severe consequences of maternal IAV infection during pregnancy, LAIV priming prior to pregnancy promoted efficacious transplacental transfer of maternal IAV-specific Ab to offspring.

## Discussion

Memory T cell responses induced by natural infection or by LAIV in mice and humans protect against heterotypic strains of IAV (19–21, 73). Whether such responses persist and retain their antiviral functions during the altered physiological state of pregnancy has not, to the best of our knowledge, been addressed previously. Since heterosubtypic T cell immunity protects against IAV infection for periods of up to a year (22–24), we reasoned that LAIV vaccination prior to pregnancy could provide a means to protect the at-risk pregnant population (74–76) against drifted and shifted strains of IAV while also overcoming the contraindication of LAIV use during pregnancy. Alternative strategies to induce universal heterosubtypic immunity in pregnant females are currently lacking (77). Our findings show that IAV-specific CD4^+^ and CD8^+^ memory T cells do indeed persist in the pregnant environment, and importantly, they retain protective antiviral effector functions.

A hallmark of protective immunity against IAV is prevention of infection-associated morbidity, which is typically evaluated in murine models as percent weight loss (78). During infection, a generalized catabolic metabolic response driven by excessive expenditure of body energy combined with reduced food and nutrient intake causes body wasting (79). Maternal weight gain during pregnancy masks wasting following IAV infection. Catabolic metabolic responses triggered by infection are, nonetheless, of great concern for the maternal-fetal unit (80, 81) as the energy and nutritional demands of pregnancy are high (69). A marked decline in the metabolic health of unprimed gravid dams following maternal IAV infection was observed in our study, but we show recall of LAIV-induced heterosubtypic T cell immunity preserves maternal metabolic and nutritional wellness that is key for healthy fetal development. These findings highlight the importance of employing alternative metrics to determine vaccine efficacy in preclinical models of maternal infection and further support that preexisting heterosubtypic T cell immunity can be beneficial for maternal health.

A major concern of relying on heterosubtypic T cell immunity to protect pregnant females from IAV is whether recall will harm the developing fetus (45, 82). Increased susceptibility during pregnancy to viral and parasitic infections normally well controlled by cell-mediated immunity only heightens concerns (83). Full-to-partial protection against the adverse congenital outcomes of cytomegalovirus and *Toxoplasma gondii* infection by preexisting maternal immunity, however, has been reported (84–86). When offspring outcomes of primary maternal IAV infection were assessed in our experiments, a high incidence of litter loss, which is prevented by LAIV vaccination prepregnancy, was observed. Moreover, preexisting maternal heterosubtypic immunity prevented the influenza-associated low birth weight that is often observed clinically following maternal IAV infection (71, 72). The latter observation is noteworthy, as the efficacy of trivalent inactivated vaccine during pregnancy in preventing low birth weight following laboratory-confirmed maternal IAV infection is, thus far, inconclusive (15). While further in-depth evaluation of the safety of the recall of IAV-specific T cell immunity during pregnancy is required, our findings support that LAIV-induced maternal heterosubtypic T cell–responses can be beneficial for fetal and newborn development.

An unanticipated benefit of maternal heterosubtypic immunity revealed here is the more efficacious transplacental transfer of maternal IAV-specific Ab to offspring. Maternally transferred Ab protects newborns against numerous pathogens in the first weeks and months of life (87–89) and the level transferred positivity correlates with the degree and longevity of passive immunity conferred (90–92). Maternal infections can compromise transplacental transfer of pathogen-specific Ab (92–95). Transfer of Ab across the placenta occurs through a receptor-mediated process involving the neonatal Fc receptor (FcRn), which preferentially binds digalactosylated Abs (96, 97) that increase in abundance during pregnancy (98). Interestingly, levels of Ab glycosylation inversely correlate with systemic inflammation and are decreased following severe infection (99, 100). The inflammatory mediators detected systemically following respiratory viral infection (101, 102) can also downmodulate FcRn expression (103). It is thus conceivable that maternal heterosubtypic T cell immunity, which effectively controls IAV and prevents severe infection, facilitates efficacious transplacental transfer of IAV-specific Ab by promoting retention of optimal levels of Ab glycosylation and FcRn expression. While the underlying mechanisms driving our observations warrant further investigation, this outcome of LAIV priming prior to pregnancy is promising for the long-term protection of infants from seasonal and pandemic IAV infection.

Finally, in agreement with LAIV-primed heterosubtypic immunity being protective, adoptively transferred IAV-specific memory CD4^+^ T cells retained the ability to control IAV during pregnancy. Primary CD4^+^ T cells responding against IAV failed to show any evidence of a pregnancy-associated shift toward unprotective T_H_2 functional response profiles (104) in both C57BL/6J and BALB/c strains of mice, the latter of which is more prone to develop stronger humoral and T_H_2 responses (105) and is more susceptible to pathogens controlled by T_H_1 responses (106, 107). Rather, expression of the T_H_1 master transcription factor T-bet was increased in primary T cells responding to IAV in gravid hosts, which is in line with prior reports of pregnancy-hormone–associated increases in IFN-γ and T-bet expression in murine T cells (108–110) and human T cell clones (111). In our studies, T-bet deficiency liberated enhanced T_H_2 responses against IAV that are known to be to be detrimental (50, 104) and likely underly the inability of T-bet–deficient immunity to prevent the adverse outcomes of maternal IAV infection. The potent inflammatory response induced by IAV (101, 112), the inaccessibility of chromatin and genes with estrogen response elements that promote T_H_2 characteristics (113–116), as well as the potential, and intriguing, differential regulation of estrogen signaling pathways in distinct CD4^+^ T cell subsets (53, 54) likely all contribute to the retention of antiviral CD4^+^ T cell responses against IAV in the pregnancy environment.

While further studies are required to discern exactly how antiviral T cell functions are retained, our findings demonstrate that LAIV vaccination prior to pregnancy induces the generation of IAV-specific T cell immunity that persists and protects the maternal-fetal unit from adverse consequences of IAV-infection. Targeting the generation of virus-specific T cell immunity against respiratory viruses of concern, such as IAV and SARS-COV-2, through vaccination prior to pregnancy has potential to protect this vulnerable at-risk population.

## Methods

### Sex as a biological variable.

Both male and female TcR Tg mice were used in adoptive transfer experiments. Only females could be used as donor cell recipients or infected with IAV.

### Mice.

BALB/c Thy1.1, HNT TcR Tg, and C57BL/6J WT and T-bet (*tbx21*^–/–^)–deficient OT-II TcR Tg mice were obtained from the Lake Nona Vivarium at the University of Central Florida. C57BL/6J and B6.SJL-Ptprca Pepcb/BoyJ (Pepboy) mice were purchased from Jackson Laboratories. HNT mice express a TcR (Va15, Vβ8.3) recognizing aa 126–138 (HNTNGVTAACHSE) of A/Puerto Rico/8/34 hemagglutinin presented on I-A^d^ (117), and OT-II Tg mice express a TcR (Va2, Vβ5) recognizing aa 323–339 (ISQAVHAAHAEINEAGR) of ovalbumin (OVA) presented on I-A^b^ (118, 119). All infected animals were at least 8 weeks old.

### Virus stocks and infections.

A/Puerto Rico/8/34 (PR8) (H1N1), originating from stocks at St. Jude Children’s Hospital (Memphis, Tennessee, USA), PR8-OVA_II_ (H1N1) (kindly provided by P. Doherty (St. Jude Children’s Hospital), and cold-adapted attenuated A/Alaska/6/77 CR-29, (H3N2) (120) (kindly provided by S. Epstein, NIH [Rockville, Maryland, USA]) viruses were grown in the allantoic cavity of embryonated hen eggs at Trudeau Institute and egg infective dose (EID_50_), tissue culture infective dose (TCID_50_), and lethal dose (LD_50_) were characterized. Mice were infected intranasally under light isoflurane anesthesia with the stated doses of virus in 50 μL of PBS. Timed-pregnant female BALB/c and C57BL/6J mice were generated following Jackson Laboratories protocols within the University of Central Florida Lake Nona Vivarium. Gravid mice, unless otherwise stated, were infected with IAV 1 week after disbanding of 3-day timed-mating cages when pregnancies could be confirmed (121). Infected mice were monitored daily for percentage weight loss, hunched posture, ruffled fur, and lack of movement and humanely euthanized as described (78).

### CD4^+^ T cell isolations, in vitro culture, and adoptive transfers.

Naive CD4^+^ T cells were obtained from pooled spleen and lymph nodes of unimmunized mice. Single-cell suspensions were passed over nylon wool and separated by Percoll (Sigma-Aldrich) density gradient centrifugation. CD4^+^ cells were further enriched employing CD4 MACS beads (Milteny Biotec). Resulting Tg cells were routinely greater-than 97% Tg TcR^+^ and expressed a characteristic naive phenotype (small size, CD62L^hi^, CD44^lo^, and CD25^lo^).

In vitro–generated memory cells were generated from thoroughly washed effectors that were recultured in fresh media for 3 days in the absence of Ag and cytokine, as previously described (122). Live cells were isolated by Lympholyte separation (CedarLane). Effectors were generated from HNT TcR Tg CD4^+^ T cells as follows: naive cells (2 × 10^5^ cells/mL) were cultured with APC (1 × 10^5^ cells/mL of CD90.1 bead–depleted [Milteny Biotec] spleen cells) in the presence of 5 μM peptide and IL-2 (80 U/mL) (Peprotech) for 4 days in RPMI 1640 media supplemented with 2 mM L-glutamine, 100 IU penicillin, 100 μg/mL streptomycin (Invitrogen), 10 mM HEPES (Gibco), 50 μM 2-mercaptoethanol (Sigma-Aldrich) and 7.5% FBS (Hyclone). T_H_1 effectors were generated by further adding IL-12 (2 ng/mL) (Peprotech) and anti–IL-4 (11B11; 10 μg/mL); T_H_2 effectors were generated by further adding IL-4 (200 U/mL) (Peprotech) and anti-IFN-γ (XMG1.2; 10 μg/mL). In some experiments, varied concentrations of 17-β estradiol (BioTechne), progesterone (BioTechne), 17-β estradiol-plus-progesterone, or vehicle control (ethanol) were added to T_H_1 and T_H_2 cultures, which were plated on the same day. Concentrations of 17-β estradiol (E2) and progesterone (P4) comparable to those found systemically during the first (0.0176 μM E2 and 0.096 μM P4), second (0.0353 μM E2 and 0.297 μM P4), and third (0.0053 μM E2 and 0.382 μM P4) trimester of pregnancy or administered pharmaceutically (1 μM of E2 and P4) were employed (52).

Naive or memory CD4^+^ T cells were adoptively transferred to recipients in 200 μL PBS by i.v. injection. In all experiments, mice received equal numbers of naive and memory CD4^+^ T cells (2–3 × 10^6^ cells as indicated) on the same day that mice were infected with IAV.

### Detection of IAV titer.

At the indicated day after infection, mice were euthanized by cervical dislocation followed by exsanguination by perforation of the abdominal aorta. Lungs were harvested and flash frozen in liquid nitrogen and viral titer was determined by quantitation of viral RNA. RNA was prepared from whole-lung homogenates using TRIzol (Invitrogen), and 2.5 μg of RNA was reverse transcribed into cDNA using random hexamer primers and Superscript II Reverse Transcriptase (Invitrogen). Quantitative PCR was performed to amplify the acidic polymerase (PA) gene of PR8 using the QuantStudio 7 Flex Real-Time PCR System (Applied Biosystems) with 50 ng of cDNA per reaction and the following primers and probe: forward primer, 5′-CGGTCCAAATTCCTGCTGA-3′; reverse primer, 5′CATTGGGTTCCTTCCATCCA-3′; probe, 5′-6-FAM-CCAAGTCATGAAGGAGAGGGAATACCGCT-3′ (LGC BioSearch Technologies). Data were analyzed with QuantStudio 7 software. The copy number of the PA gene per 50 ng of cDNA was calculated using a PA-containing plasmid of known concentration as a standard. The number of copies of the PA gene is presented as the number of IAV copies per lung.

### Tissue preparation and flow cytometry.

At the indicated time points following virus infection, mice were euthanized by cervical dislocation, exsanguinated by perforation of the abdominal aorta, and lungs perfused by injecting 10 mL of PBS in the left ventricle of the heart. Lungs, spleen, and dLN were prepared into single cell suspensions by mechanical disruption and passage through a nylon membrane. Cell suspensions were washed, resuspended in FACS buffer (PBS plus 0.5% BSA and 0.02% sodium azide (NaN_3_) [Sigma-Aldrich]) and incubated on ice with 1 μg anti-FcR (2.4G2, BioXcell) followed by saturating concentrations of the following fluorochrome-labeled Abs for surface staining: anti-Thy1.1 (OX-7), -Thy1.2 (53-2.1), -CD4 (RM4.5), -CD8 (H35-17.2), -CD44 (IM7), and-CD45.2 (104), (BD Biosciences, eBioscience/Thermo Fisher Scientific, or BioLegend). To enumerate IAV-specific polyclonal CD4^+^ and CD8^+^ T cells in IAV-primed mice, cells were stained for 1 hour at 37°C with I-A^b^/NP_311-325_-fluorochrome–labeled tetramer (NIH tetramer facility) or for 1 hour at room temperature with H2D^b^ PA_224-233_ fluorochrome–labeled tetramer (NIH tetramer facility), respectively, prior to surface marker staining.

For intracellular cytokine staining (ICCS), cells were stimulated for 4 hours with 10 ng/mL PMA and 50 ng/mL ionomycin (Sigma-Aldrich). After 2 hours, 10 μg/mL Brefeldin A (Sigma-Aldrich) was added. Cells were then surface stained and fixed for 20 minutes in 4% paraformaldehyde followed by permeabilization for 10 minutes by incubation in 0.1% saponin buffer (PBS plus 1% FBS, 0.1% NaN3 and 0.1% saponin [Sigma-Aldrich]) and staining for cytokine by addition of anti–IFN-γ (XMG1.2), –IL-2 (JES6-5H4), -TNF (MP6-XT22), –IL-17 (TC11-18H10), –IL-4 (11B11), –IL-5 (TRFK5), –IL-13 (eBio13A), and –GM-CSF (MP1-22E9) fluorescently labeled Ab (BD Biosciences, eBioscience, or BioLegend) for 20 minutes. Intracellular staining for transcription factors was performed per the manufacturer’s instructions with Transcription Factor Fixation/Permeabilization Concentrate and Diluent (eBioscience/Thermo Fisher Scientific) and fluorochrome-labeled anti–T-bet (4B10), -EOMES (Dan11mag), and –GATA-3 (TWAJ) Ab (BD Biosciences, eBioscience, or BioLegend). Prohibitin-2 staining was also performed using the Transcription Factor Fixation/Permeabilization Concentrate and Diluent (eBioscience) with primary anti–Prohibitin-2 (Poly6118) Ab (BioLegend) followed by secondary PE-labeled donkey anti-rabbit IgG (Poly4064) Ab (BioLegend).

To detect estrogen receptor α (ERα) expression in different cellular compartments (123, 124), separate effector cell suspensions were prepared for surface, intracellular (cytosolic), and transcription factor (nuclear) staining as described above and stained with primary anti-ERα Ab (NR3A1) (R&D systems) followed by secondary PE-labeled donkey anti-rabbit IgG (Poly4064) Ab. Analysis was performed using FACS Canto II (BD Biosciences) and Cytoflex (Beckman Coulter) instruments and FlowJo (Tree Star) analysis software. Spice 6 software (NIH) was used to visualize single, double, and triple cytokine production (125).

### ELISA detection of cytokines and IAV-specific Ab.

In vitro polarized T_H_1 or T_H_2 effectors were harvested and restimulated with immobilized anti-CD3 (2C11, BioXcell) and anti-CD28 (37.51, BioXcell), as previously described (122). Culture supernatants from triplicate wells were collected after 24 hours. ELISA assays using anti–IL-4 (11B11) and anti–IFN-γ (R4-6A2) as coating Abs and biotinylated-anti–IL-4 (BVD6-24G2) and anti-IFN-γ (XMG1.2) as second step reagents (BD Biosciences) were employed to determine levels of IL-4 and IFN-γ in supernatants. After addition of streptavidin-HRP conjugate and washing, HRP substrate *o*-phenylenediamine dihydrochloride (Sigma-Aldrich) was added and the optical density (OD) of the color reaction was measured at 492 nm. Concentrations of cytokines were quantitated from standard curves using recombinant cytokines (BD Biosciences).

Reciprocal endpoint titers of PR8-specific IgG Ab of infected dams and their offspring at weaning were determined by ELISA. Briefly, ELISA plates (Nunc) coated with UV-inactivated PR8 were washed and blocked with PBS containing 2% BSA. Serum samples serially diluted in PBS-Tween 20 with 1% BSA were incubated for 2 hours at room temperature. After washing, HRP-conjugated goat anti-mouse IgG (1030-05, Southern Biotech) was added at 0.2 μg/mL in PBS-Tween 20 with 1% BSA, and plates were incubated 1 hour at room temperature. After washing, the HRP substrate *o*-phenylenediamine dihydrochloride (Sigma-Aldrich) was added and the OD of the color reaction was measured at 492 nm. Endpoint serum titers were defined by the last serum dilution that gave OD readings above 2 SD of the mean of conjugate blank readings.

### Metabolic and behavioral monitoring.

Indirect calorimetry and physical activity data were monitored using the Promethion Cage Monitoring System (Sable Systems International). Oxygen consumption, carbon dioxide production, food and water intake, and infrared beam breaks as a measure of pedestrian locomotion from individually housed animals were collected at 5 minute intervals for 6 days following IAV infection. Animals were briefly acclimatized to cages prior to random assignment to cages for data collection. Experiments were completed prior to anticipated parturition of pregnant dams. CalR was used to generate hourly and cumulative data plots from imported raw data (70).

### Histology.

For assessment of immunopathology following IAV challenge infection, lungs lobes were isolated and immediately fixed in 10% neutral buffered formalin (Thermo Fisher Scientific) on 5 days after infection. Lung lobes were embedded in paraffin, sectioned at 5 μm thickness, placed on L-lysine–coated slides, and stained with H&E using standard histological techniques. Sections were graded blindly from 0 to 4 by a board-certified pathologist (S. Sell, Palisades Consulting, Williamsburg, Virginia, USA) based on the extent of mononuclear cell infiltration and tissue damage. Stitched Z-stack images of whole lung lobe sections at 20 × magnification were acquired with the ImageXpress Pico Automated Cell Imaging System (Molecular Devices) and CellReporterXpress software (V 2.7).

### Morphological development of newborn mice.

Body weight and measurements of crown rump length, bizygomatic width, and femur length at the indicated time points were used to monitor the development of newborn mice (126, 127). Digital refractive and X-ray images of anesthetized pups placed in prone and lateral positions within a warmed chamber were captured using the Bruker In-Vivo Xtreme Imaging System with Molecular Imaging Software. Measurements were determined with ImageJ (NIH) software.

### Statistics.

Group sizes of *n* = 3–6 per experiment were employed in repeated experiments. GraphPad Prism was used to perform statistical tests. Unpaired 1-tailed Students *t* test, μ = 0.05, was used to assess whether the means of 2 normally distributed groups differed, unless otherwise indicated as 2-tailed. Welch’s correction was applied when variances were found to differ. 1-way ANOVA analysis with the appropriate multiple comparison post test was employed to compare multiple means. The Log Rank test was used to test for significant differences in Kaplan-Meier survival curves. CALR was used to perform ANOVA and ANCOVA tests on raw metabolic health parameter data. All error bars represent the SD. *P* values below 0.05 are considered statistically significant.

### Study approval.

All experimental animal procedures were conducted in accordance with guidelines outlined by the Office of Laboratory Animal Welfare (OLAW), National Institute of Health, USA. Protocols were approved by the Institutional Animal Care and Use Committee of the University of Central Florida (Orlando, Florida, USA).

### Data availability.

Underlying data is available in the Supplemental Data Values file. All underlying raw data files are available directly from the corresponding author.

## Author contributions

VFM, KD, AS, ACA, LAK, AJB, SNA, and TMS participated in data collection and analysis. VFM, KD, KKM, and TMS performed experiments. KKM contributed to manuscript editing. TMS conceived, designed, and supervised the project and wrote the manuscript.

## Supplementary Material

Supplemental data

Supporting data values

## Version 1 01/02/2025

Electronic publication
