# Supplementary material for: Preexisting vaccine-primed heterosubtypic T cell immunity protects the maternal-fetal unit from adverse influenza outcomes in mice
Source: J Clin Invest. 2025 Jan 2;135(1):e179230. doi: 10.1172/JCI179230 (PMC11684801; doi:10.1172/JCI179230)
Supplement: Supplemental data [file jci-135-179230-s219.pdf]

Supplemental Data for:

Pre-existing vaccine-primed heterosubtypic T-cell immunity protects the maternal-fetal unit from adverse influenza outcomes in mice

Valeria Flores Malavet[1], Kunal Dhume[1], Ali Satchmei[1], Andrea C. Arvelo[1], Aaron J. Beaird[1], Siva N. Annamalai[1], Lauren A. Kimball[1], K. Kai McKinstry[1], and Tara M. Strutt[1, 2]

[1]Immunity and Pathogenesis Division, Burnett School of Biomedical Sciences College of Medicine, University of Central Florida, Orlando, Florida, USA

Running title: IAV-specific memory T cells protect during pregnancy

[2]Corresponding author:
Tara M. Strutt
6900 Lake Nona Blvd
Orlando, FL 32827
Email: tara.strutt@ucf.edu
Phone: 407-266-7144

Conflict-of-interest statement:
The authors have declared that no conflict of interest exists

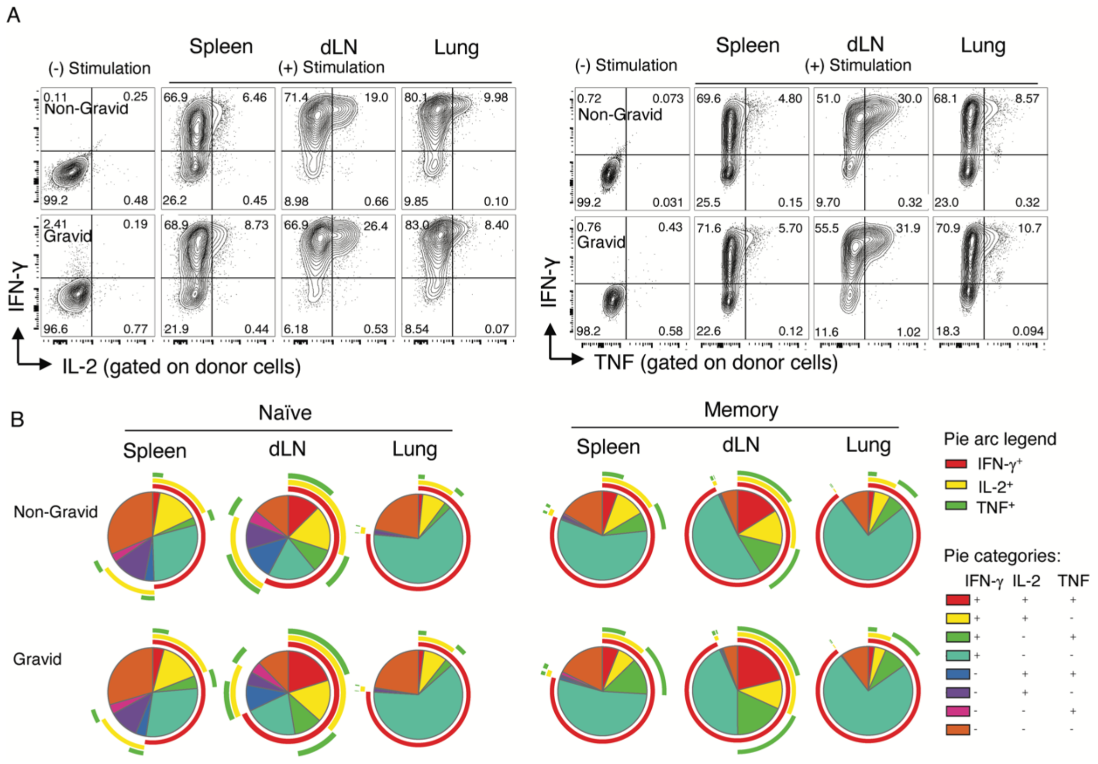

Supplemental Figure 1. *Primary and secondary T_H1 CD4 T cell cytokine responses against IAV are unaltered by the pregnancy environment.* Congenic HNT CD4 TcR Tg naïve or memory CD4 T cells, 3 x 10^6, were adoptively transferred to unprimed non-gravid or timed-pregnant BALB/c recipients that were infected on the same day with a pathogenic 0.5 LD_{50} PR8. All gravid dams were at the transition between the first and second trimester of pregnancy when infected. On 7 dpi, T_H1-associated single, double, and triple cytokine production by effector cells in the spleen, dLN, and lung of recipients was characterized by flow cytometry and ICCS (n = 3-4 mice per group in replicate experiments). Representative staining for IFN-γ, IL-2, TNF is shown in (**A**). The average (**B**) proportion of primary or secondary CD4 T effector cells in each organ producing one, two, or three cytokines is shown in Spice pie charts with arcs depicting the total production of each cytokine.

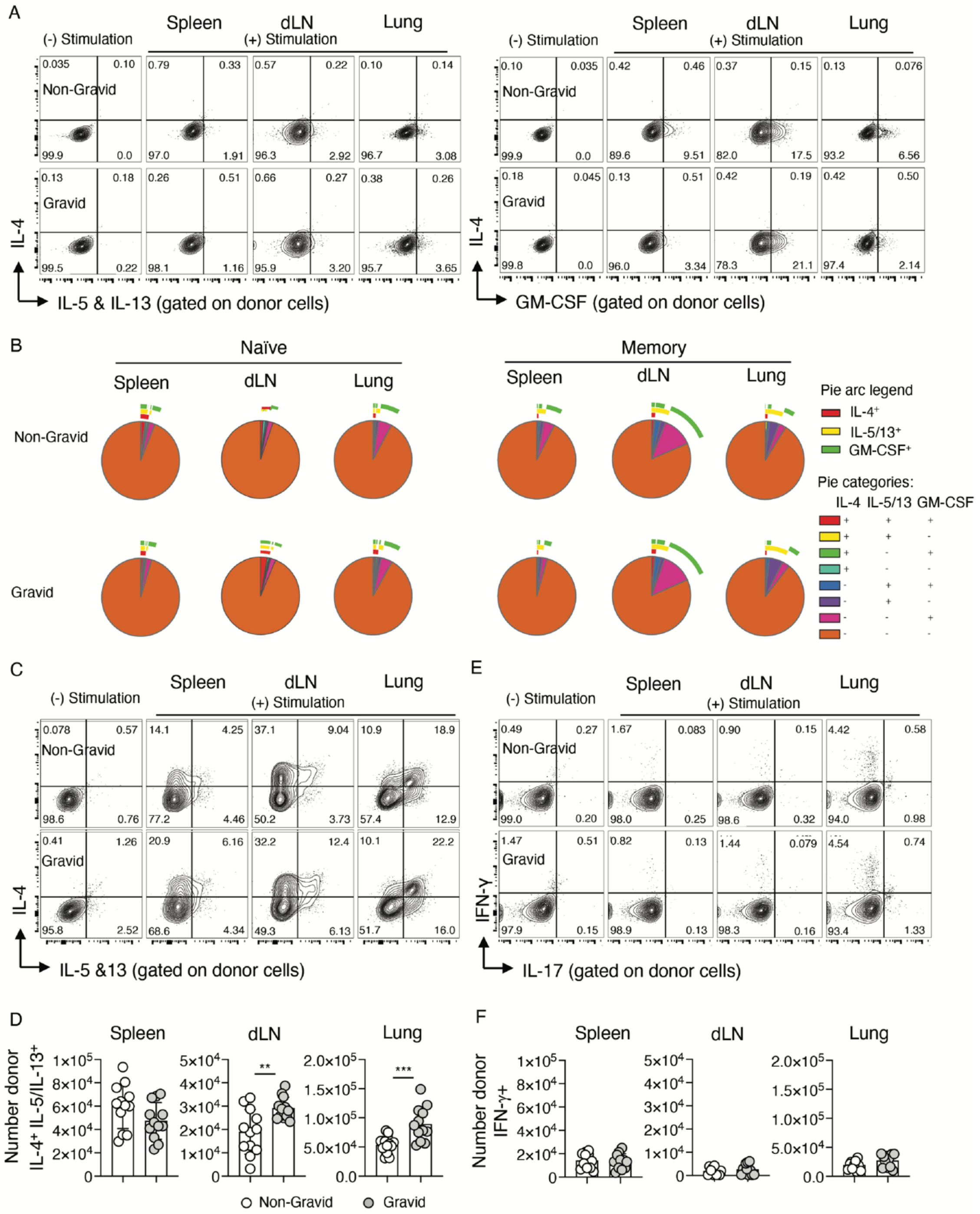

Supplemental Figure 2. *Primary and secondary anti-viral effector CD4 T cells fail to shift towards T_H2-associated cytokine production in the pregnancy environment.* Congenic HNT CD4 TcR Tg naïve or memory CD4 T cells, 3 x 10^6, were transferred to unprimed non-gravid or timed-

pregnant BALB/c female recipients that were infected on the same day with a pathogenic 0.5 LD_{50} PR8 as described in Supplemental Figure 1. On 7 dpi, T_H2-associated single, double, and triple cytokine production by effector cells in the spleen, dLN, and lung of recipients was characterized by flow cytometry and ICCS (n = 3-4 mice per group in replicate experiments). Representative staining for IL-4, IL-5 & IL-13, and GM-CSF is shown in (**A**). The average (**B**) proportion of naïve and memory donor derived CD4 T effector cells in each organ producing one, two, or three cytokines is shown in Spice pie charts with arcs depicting the total production of each cytokine. In separate experiments, 3 x 10^6, congenic T_H2 HNT CD4 TcR Tg effector T cells were adoptively transferred to unprimed non-gravid or timed-pregnant BALB/c female recipients infected with PR8 as above. On 7 dpi, T_H2-associated cytokine production by effector cells in the spleen, dLN, and lung of recipients was characterized by ICCS (n = 3-4 mice per group in replicate experiments). Representative staining and enumeration (**C** and **D**) of donor IL-4-, IL-5- and IL-13-producing cells as well as (**E** and **F**) IFN-γ and IL-17 production and enumeration of IFN-γ producing cells in each organ. Students *t*-tests were used for pairwise comparisons in D and E.

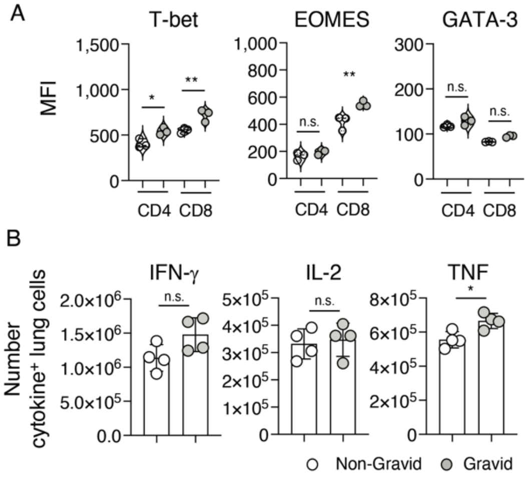

Supplemental Figure 3. *T_H1-associated transcription factor expression is increased in endogenous CD4 and CD8 T cells responding against primary IAV infection in gravid mice.* Non-gravid or timed-pregnant BALB/c female recipients were infected with 0.5 LD_{50} PR8 at the transition between the first and second trimester of pregnancy. On 7 dpi, $CD44^{hi}$ CD4 and CD8 T cells in the lung were assessed for T-bet, EOMES, and GATA-3 expression by flow cytometry and (**A**) MFI values determined (n = 3 per group, representative of 2 separate experiments). On 7 dpi, T_H1-associated cytokine production by endogenous T cells was characterized by ICCS and (**B**) the number of IFN-γ–, IL-2-, and TNF-producing cells in the lung enumerated (n = 4 mice per group in 2 replicate experiments). Students *t*-tests were used for pairwise comparisons in A and B.

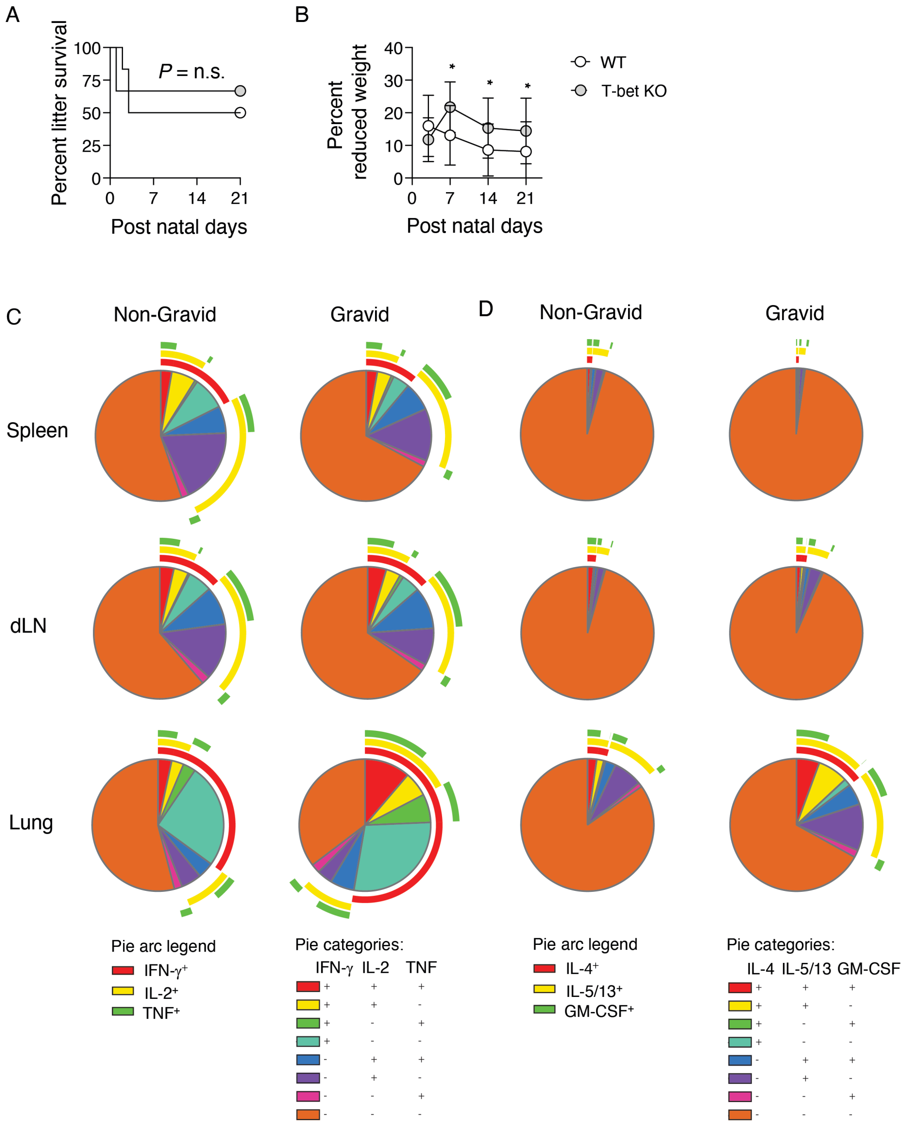

Supplemental Figure 4. *T-bet deficient immunity is altered during pregnancy and fails to protect the maternal-fetal unit from the adverse outcomes of IAV.* C57BL/6 WT and T-bet deficient (KO) gravid mice were infected with 0.5 LD_{50} PR8 at the transition between the first and second trimester of pregnancy. Following parturition, litter (**A**) survival and (**B**) pup postnatal weights were monitored (n = 3 dams per group and n = 18 to 22 pups per group, summation of 2 separate experiments). The postnatal weights of pups born to uninfected, and strain matched dams were used in calculations to determine the percent reduced weight. In separate experiments, naïve

congenic T-bet deficient OT-II CD4 TcR Tg CD4 T cells, 2 x 10^6, were transferred to unprimed non-gravid or timed-pregnant WT recipients that were subsequently infected with 0.2 LD_{50} PR8-OVA_{II}. On 7 dpi, T_H1-and T_H2-associated single, double, and triple cytokine production by effector cells in the spleen, dLN, and lung of recipients was characterized by flow cytometry and ICCS (n = 4 mice per group in replicate experiments). Spice pie charts showing the average proportion of donor derived CD4 T effector cells in each organ producing one, two, or three cytokines with arcs depicting the total production of each cytokine for (**C**) T_H1-, and (**D**) T_H2-associated cytokines. The Log Rank test was used in A, and ordinary two-way ANOVA with repeated measures and uncorrected Fisher's LSD post-tests employed in B.
